# Gamma-secretase: from pathogenesis to therapeutics

**DOI:** 10.1186/1750-1326-7-S1-L4

**Published:** 2012-02-07

**Authors:** Yueming Li

**Affiliations:** 1 Molecular Pharmacology and Chemistry Program, Memorial Sloan-Kettering Cancer Center and Department of Pharmacology, Weill Graduate School of Medical Sciences of Cornell University, New York, USA

## Background

Presenilin (PS) is the catalytic subunit of γ-secretase and mutations in this protein cause familial Alzheimer’s Disease (FAD). Moreover, γ-secretase has emerged as an appealing drug target for Alzheimer’s disease (AD) and cancer due to its central role in the generation of Aβ peptides and the regulation of Notch signaling. γ-Secretase is composed of at least four subunits: PS, Nicastrin, Aph1 and Pen2; with a total of 19 putative transmembrane domains.

## Result

Investigation of γ-secretase structure and function has been a formidable challenge because of its nature of intramembranal catalysis and macromolecular complex that requires novel chemical insights. We have developed a reconstituted system and small molecular probes that allow us to study the regulation of γ-secretase and to elucidate the mechanism of PS1 FAD mutations. We have reconstituted γ-secretase using a proteoliposomes system and provided the final proof that γ-secretase activity is an inherent property of PS. Moreover, we have demonstrated that PS mutations directly alter a subsite of γ-secretase active site and defined the action mechanism of γ-secretase modulators.

## Conclusion

Our studies provide a molecular basis of PS1 mutations in AD pathogenesis and offer a unique approach to elucidate the reaction mechanism of γ-secretase, with the expectation that these efforts will lead to the development of effective therapies for AD and other human disorders.

